# Decreases in cerebral saturation in patients with septic shock are associated with increased risk of death: a prospective observational single center study

**DOI:** 10.1186/s40560-016-0167-y

**Published:** 2016-06-29

**Authors:** Duane J. Funk, Anand Kumar, Gregory Klar

**Affiliations:** University of Manitoba, Winnipeg, Canada; Department of Medicine, Section of Critical Care, College of Medicine, Faculty of Health Sciences, University of Manitoba, Winnipeg, Canada

**Keywords:** Septic shock, Cerebral oxygen saturation, Monitoring

## Abstract

**Background:**

The mortality rate from septic shock has been declining. Cerebral hypoxia, measured non-invasively with cerebral oximetry, has been correlated with neurologic and non-neurologic sequelae. Whether cerebral desaturations occur in septic shock patients and what consequences these may have is untested.

**Methods:**

Adult patients with septic shock had cerebral saturation monitoring initiated. The primary objective was to determine if the incidence and magnitude of cerebral desaturations in septic shock patients correlated with delirium. We also compared the incidence and magnitude of cerebral desaturations in patients with septic shock with patients undergoing high-risk non-cardiac surgical procedures, a group known to be at high risk for cerebral desaturations.

**Results:**

Fifteen patients were enrolled. Twelve (80 %) patients had a decrease in SctO_2_ below 65 %. Delirium was not associated with the area under the curve of an SctO_2_ of 65 % (*p* = 0.84).

Patients who died of septic shock had more significant decreases in SctO_2_ than those who survived (*p* = 0.04).

Decreased SctO_2_ was more common in patients with septic shock and was of greater magnitude than those undergoing high-risk non-cardiac surgery.

**Conclusions:**

Cerebral desaturations occur more commonly and are of a greater magnitude in septic shock patients compared with those undergoing high-risk non-cardiac surgery. There did not appear to be a relationship between the incidence or magnitude of decreases in SctO_2_ and ICU delirium. Patients who died of septic shock had more significant decreases in SctO_2_ than patients who survived.

## Background

Over the past 15 years, the mortality rate from severe sepsis and septic shock has been declining [1–5]. With this decreased mortality rate, clinicians are now focusing increasing efforts on reducing sepsis-related morbidity, including intensive care unit (ICU)-acquired delirium.

Delirium in the ICU is a prevalent problem, occurring in up to 80 % of patients [6]. The etiology is thought to be multifactorial including the effects of lack of sleep, noise in the ICU, use of sedatives and analgesics, and sepsis related to infection [7–9]. Cerebral hypoxia could be a potential cause of delirium in ICU patients. There is emerging data from the anesthesia literature suggesting that occult intraoperative cerebral hypoxia results in postoperative morbidity, including postoperative cognitive dysfunction [10–13]. These decreases in brain tissue oxygen saturation (SctO_2_) can be measured non-invasively with cerebral oximetry. In the operative environment, cerebral desaturations usually occur in the absence of systemic hypoxemia [13]. The question of whether cerebral desaturations occur in ICU patients (and in particular, septic shock patients) and what, if any, consequences these may have is untested.

During the resuscitative phase of septic shock, a low cardiac output state occurs. During these low flow states, the regional distribution of blood flow to vital organs such as the brain occurs at the expense of other tissue beds such as the kidney and splanchnic circulation [14, 15]. A reliable non-invasive continuous perfusion monitor has not been utilized in patients with shock.

In this single-center prospective observational trial, our primary objective was to determine if the incidence and magnitude of cerebral desaturations in septic shock patients correlated with delirium, as measured by the Confusion Assessment Method for the Intensive Care Unit (CAM-ICU) score [16]. Secondary outcomes examined were the association between the incidence and magnitude of cerebral desaturations and acute kidney injury (AKI) or death (up to 28 days after the diagnosis of sepsis). We also compared the incidence and magnitude of cerebral desaturations in patients with septic shock with patients undergoing high-risk non-cardiac surgical procedures, a group known to be at high risk for cerebral desaturations and high risk for complications [10, 17].

## Methods

This trial was registered at Clinicaltrials.gov as NCT1836302. After approval by the University of Manitoba Biomedical Research Ethics Board, we recruited all patients over the age of 18 years who had been admitted to either the Medical or Surgical/Trauma ICU at the University of Manitoba Health Sciences Center with the diagnosis of severe sepsis and septic shock for a 6-month period. Written consent for inclusion in the study was obtained from the patient’s family on a deferred basis.

The definition of severe sepsis and septic shock was based on the American College of Chest Physicians/Society of Critical Care Medicine Consensus Conference Committee [18]. Patients were treated according to the Surviving Sepsis Guidelines with early antimicrobial therapy and source control, aggressive fluid resuscitation, mechanical ventilation, and hemodynamic support [19].

Patients with severe sepsis and septic shock had bilateral near-infrared spectroscopic cerebral oximiter sensors (Foresight®, CASMED, Brandford, CT, USA) placed on their forehead. Sensors were placed within 12 h of the patients being admitted to the ICU in order to capture any desaturations that might occur during the resuscitative phase of their septic shock. Sensors were kept in place for at least 24 h, with a maximum monitoring time of 48 h. If a patient regained hemodynamic stability (as defined as no longer needing vasopressor support) after 24 h, monitoring was discontinued. The monitoring was also discontinued and reinstituted in order to facilitate travel for diagnostic imaging or other procedures outside the ICU.

Cerebral saturation data were collected continuously at 0.5 Hz. After the study period ended, the data were downloaded from the monitor and placed into a Microsoft Excel (Redmond, WA) spreadsheet and the values were averaged on an hourly basis. Hemodynamic values and vasopressor doses were recorded hourly.

Patients were sedated to a RASS goal of −1 to −2 as per institutional protocol (typically with a combination of either propofol, fentanyl, or midazolam infusions). The CAM-ICU assessment was measured twice daily for the duration of ICU stay. Daily interruption of sedation was not performed. The CAM-ICU assessment occurred for several more days after SctO_2_ monitoring was discontinued in an attempt to capture the potential for delayed effects of decreases in SctO_2_ on the incidence of delirium.

Baseline demographic data on each patient was collected. In addition to SctO_2_, levels, other patient variables that were collected included hourly blood pressure, hourly vasopressor requirements, fraction of inspired oxygen, and peripheral oxygen saturation. Sequential organ failure assessment (SOFA) and the Acute Physiology and Chronic Health Evaluation II (APACHE II) scores were also collected for each patient.

Outcome variables measured included the presence of delirium as determined by a positive Confusion Assessment Method for the Intensive Care Unit (CAM-ICU) score, acute kidney injury (using the AKIN criteria [20]), the need for two or more vasopressors/inotropes, and ICU mortality.

As a comparator group, the incidence and magnitude of cerebral desaturations was compared with data from a concurrent trial of patients undergoing high-risk non-cardiac surgical procedures utilizing the same monitor (NCT01838733). This is a patient group that is known to be at high risk for cerebral desaturations [10, 17].

### Availability of data and materials

Our research ethics board does not allow the release of databases without consent. These will not be available to readers.

### Statistical analysis

As the definition of cerebral desaturation varies from study to study, we examined several exploratory markers of desaturation including overall number of desaturations (to less than < 65 %), nadir cerebral saturation (SctO_2_), percent time under threshold (defined as the percentage of time spent below an SctO_2_ level of 65 %, to account for variable recording lengths between patients), and the area under threshold (AUT; a measurement that takes into account the time below a threshold, as well as the magnitude of the decrease in SctO_2_, units of %-min). Values of AUT were divided by the total recording time for patients to obtain values on a per hour basis to take into account the varied recording times.

In examining the relationship between changes in hemoglobin level and SctO_2_, we examined various SctO_2_ thresholds at 5 % intervals (60–75 %) to determine which cutoff had the highest correlation with change in day 2 hemoglobin.

As this was a pilot study, we calculated our sample size based on the method of Viechtbauer et al. [21]. A previous work in high-risk surgical patients has demonstrated a cerebral desaturation rate of 20 % [10, 12, 17, 22, 23]. Assuming a similar incidence in patients with septic shock, we determined that studying 14 patients would give us 95 % confidence to detect a 20 % probability of desaturations.

Statistical analysis was performed using GraphPad Prism version 6.0 (GraphPad Software Inc., La Jolla, CA, USA). Categorical variables were analyzed with the Fisher exact test. Between group continuous variables were analyzed with a Student *t* test or a Mann-Whitney *U* test, depending on the distribution of the data. The Kolmogorov-Smirnov test was performed to assess for normality. Continuous data are presented as mean ± standard deviation or median [interquartile range]. Correlations between SctO_2_ and SOFA and APACHE II scores were compared using a Spearman correlation test. A *p* value of 0.05 was considered significant.

## Results

During our 6-month pilot trial, we enrolled 15 patients with septic shock (see Tables 1 and 2). The average age of the patients was 57 ± 14 years. Median [interquartile range] for SOFA and APACHE II scores were 15 [12–19] and 23 [18–26], respectively. Pneumonia was the most common cause of sepsis, occurring in seven out of the 15 (47 %) patients. Eight of the 15 patients (53 %) had a causative organism identified (see Table 1). Thirteen of the patients (87 %) were mechanically ventilated and all were receiving vasopressor support with norepinephrine. Ten (67 %) were also receiving concomitant therapy with vasopressin. Seven of the 15 patients (47 %) were CAM-ICU positive, and 10 developed an AKI. There were four deaths in the septic shock group.

**Table 1:** Baseline data for the patients with septic shock

Patient	PMHx	Source of sepsis	Organism	Outcome	CAM	AKI	SOFA	APACHE	Mechanical ventilation
1	EtOH abuse	Lung	None recovered	Dead	CAM+	Yes	23	24	Yes
2	HIV, IHD	Lung	*Streptococcus pneumoniae*	Alive	CAM−	Yes	15	28	Yes
3	CHF	Lung	None recovered	Alive	CAM+	Yes	17	24	Yes
4	None	Heart/lung	*Staphylococcus aureus*	Alive	CAM+	Yes	18	19	Yes
5	IVDU, Hep C	Bacteremia	GNB	Dead	CAM+	Yes	22	26	Yes
6	None	Abdomen	None recovered	Alive	CAM+	No	10	15	Yes
7	Pancreatitis, EtOH, DM2	Abdomen	None recovered	Alive	CAM−	No	8	16	Yes
8	Smoker, Asthma	Lung	None recovered	Alive	CAM−	No	15	18	Yes
9	DM2, HTN, IHD, UGIB	Lung	*Pseudomonas* sp.	Alive	CAM−	Yes	15	30	Yes
10	None	Lung	None recovered	Alive	CAM−	No	6	9	No
11	DM2, CMP, COPD	Ischemic foot	*S. aureus*	Dead	CAM+	Yes	20	26	Yes
12	CHF, CRI, RA	Necrotizing fasciitis	Pseudomonas	Dead	CAM−	Yes	19	23	Yes
13	DM2, PVD	Leg	β-hemolytic strep	Alive	CAM−	Yes	13	21	Yes
14	Developmental delay	Abdomen	Non-hemolytic Streptococcus	Alive	CAM−	Yes	12	27	Yes
15	Autoimmune hepatitis, portal HTN, ESRD	Ankle	None recovered	Alive	CAM+	No	16	23	No

*PMHx* past medical history, *CAM*-*ICU* confusion assessment method, *AKI* acute kidney injury, *EtOh* ethanol, *HIV* human immunodeficiency virus, *IHD* ischemic heart disease, *CHF* congestive heart failure, *IVDU* intravenous drug use, *HEP C* hepatitis C virus, *DM2* type 2 diabetes, *HTN* hypertension, *UGIB* upper gastrointestinal bleed, *CMP* cardiomyopathy, *COPD* chronic obstructive pulmonary disease, *CRI* chronic renal insufficiency, *RA* rheumatoid arthritis, *PVD* peripheral vascular disease, *ESRD* end-stage renal disease

**Table 2:** Baseline demographics, co-morbidities, and type of surgery performed for the septic and surgical patients

Baseline demographics		
	Septic patients	Surgical
Number studied	15	30
Age	56.9 ± 14.0	71.4 ± 6.4
Male	6/15	20/30
Co-morbidities		
Hypertension	4	18
Angina	0	4
Previous myocardial infarct	1	7
Congestive heart failure	3	0
Ethanol abuse	2	0
HIV infection	1	0
Hepatitis C infection	1	0
Diabetes	4	5
Chronic renal insufficiency	2	1
Chronic obstructive pulmonary disease	2	2
Type of surgery performed		
Vascular		9
General surgery		6
Thoracic		13
Urologic		2

Patients in the septic shock group were monitored for an average of 39.8 ± 20.4 h. Twelve (80 %) of the septic shock patients had a decrease in their SctO_2_ to below 65 %. Six had decreases in their SctO_2_ to below 60 %.

There was no difference between the CAM-ICU positive and CAM-ICU negative groups with respect to the percentage of time spent below an SctO_2_ of 65 % nor the area under a threshold SctO_2_ of 65 % (see Table 3, all *p* values > 0.05).

**Table 3:** Relationship between magnitude of cerebral desaturation and primary and secondary outcomes

Outcome	Outcome present	Outcome absent	*p* value
CAM positive			
% Time	0.0 [0.0–1.5]	0.0 [0.0–0.3]	0.79
AUT/h	2.9 [0.2–8.4]	9.3 [1.2–16.0]	0.41
AKI			
% Time	2.0 [0.7–6.3]	2.1 [0.8–21.5]	0.51
AUT/h	2.9 [0.2–8.4]	12.6 [0.3–21.2]	0.59
Death			
% Time	0.9 [0.0–7.2]	0.0 [0.0–0.3]	0.43
AUT/h	4.5 [0.0–25.3]	0.0 [0.0–0.6]	0.04

There was no difference in SctO_2_ between groups with respect to the presence of acute kidney injury (AKI) nor being Confusion Assessment Method (CAM-ICU) positive. Patients who died had decreases in SctO_2_ of greater magnitude than those that survived. Data are presented as median [interquartile range]. Values are represented as median [interquartile range]

*% Time* percentage of time spent below an SctO_2_ of 65 %

*AUT* area under threshold (% min^−1^ hr^−1^)

There was also no difference in the percentage of time spent below an S_ct_O_2_ of 65 % in patients who did and did not suffer an AKI. The AUT of an S_ct_O_2_ < 65 % was also not different between the patients with and without an AKI (Table 3).

**Fig. 1:**
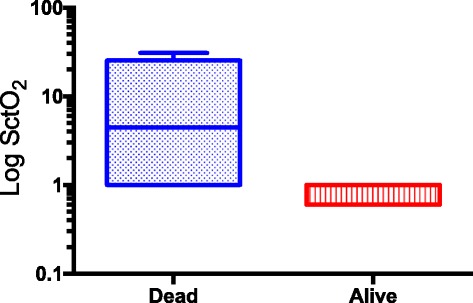
Graphical representation of the Log AUT SctO_2_ of patients who died and survived their septic shock. Patients who died had a higher median AUT S_ct_O_2_ < 65 % than patients who survived (4.5 [0.0–25.3] vs. 0.0 [0.0–0.6] % min^−1^ hr^−1^; *p* = 0.04)

Patients who died during the study, however, had a lower median AUT S_ct_O_2_ < 65 % than patients who survived (4.5 [0.0–25.3] vs. 0.0 [0.0–0.6] % min^−1^ hr^−1^; *p* = 0.04; Table 3 and Fig. 1). There was no difference in the percentage time spent under a threshold of 65 % between patients who survived and died, suggesting that it is the magnitude of desaturation that plays an important role in this outcome (Table 3).

The incidence or magnitude of decrease in SctO_2_ was not related to the severity of the patient’s illness, as measured by SOFA or APACHE II scores (data not shown).

**Fig. 2:**
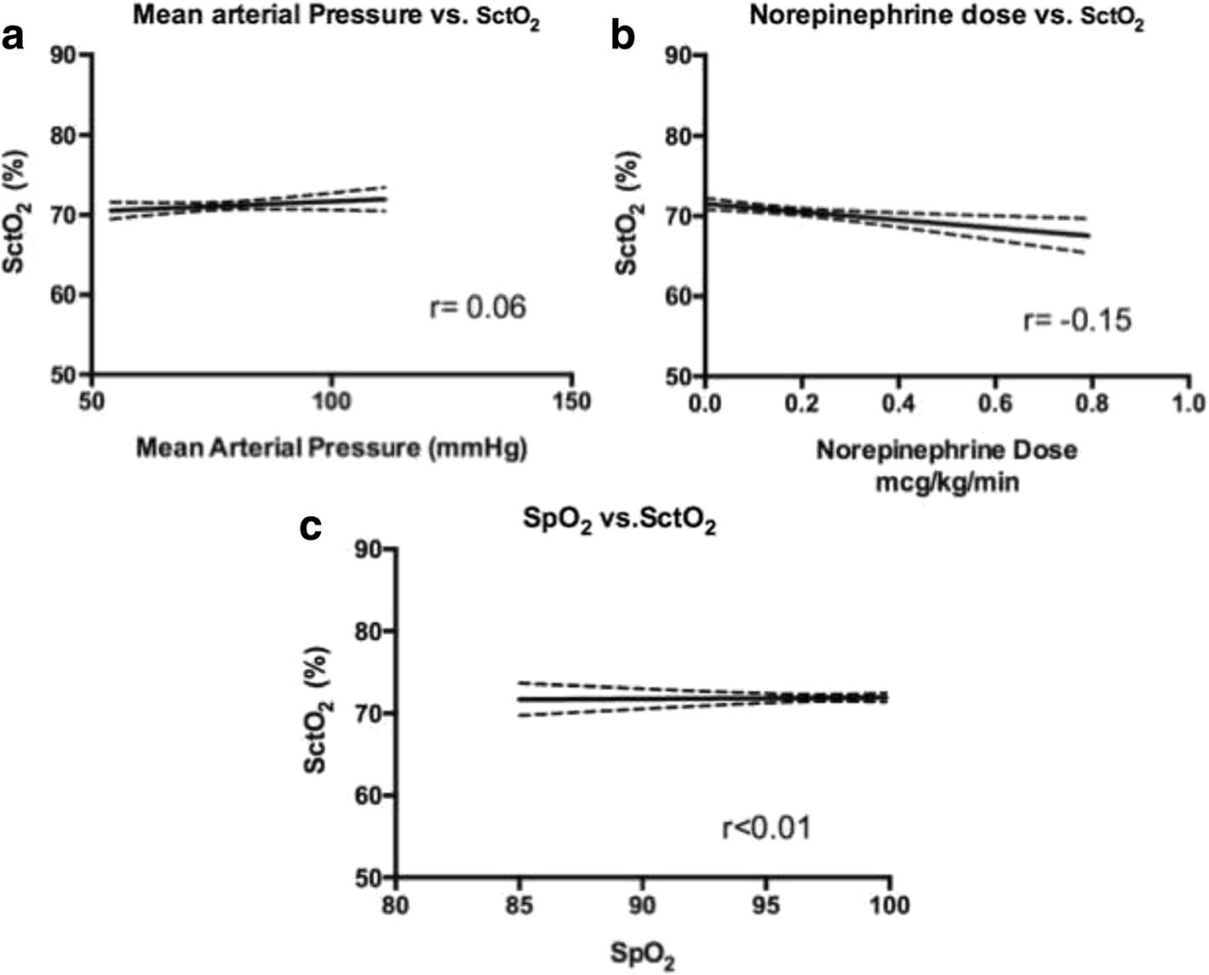
Correlation between SctO_2_ values and **a** mean arterial pressure, **b** norepinephrine dose, and **c** peripheral oxygen saturation. There was no correlation between any of these variables and SctO_2_ (Pearsons *r*)

**Fig. 3:**
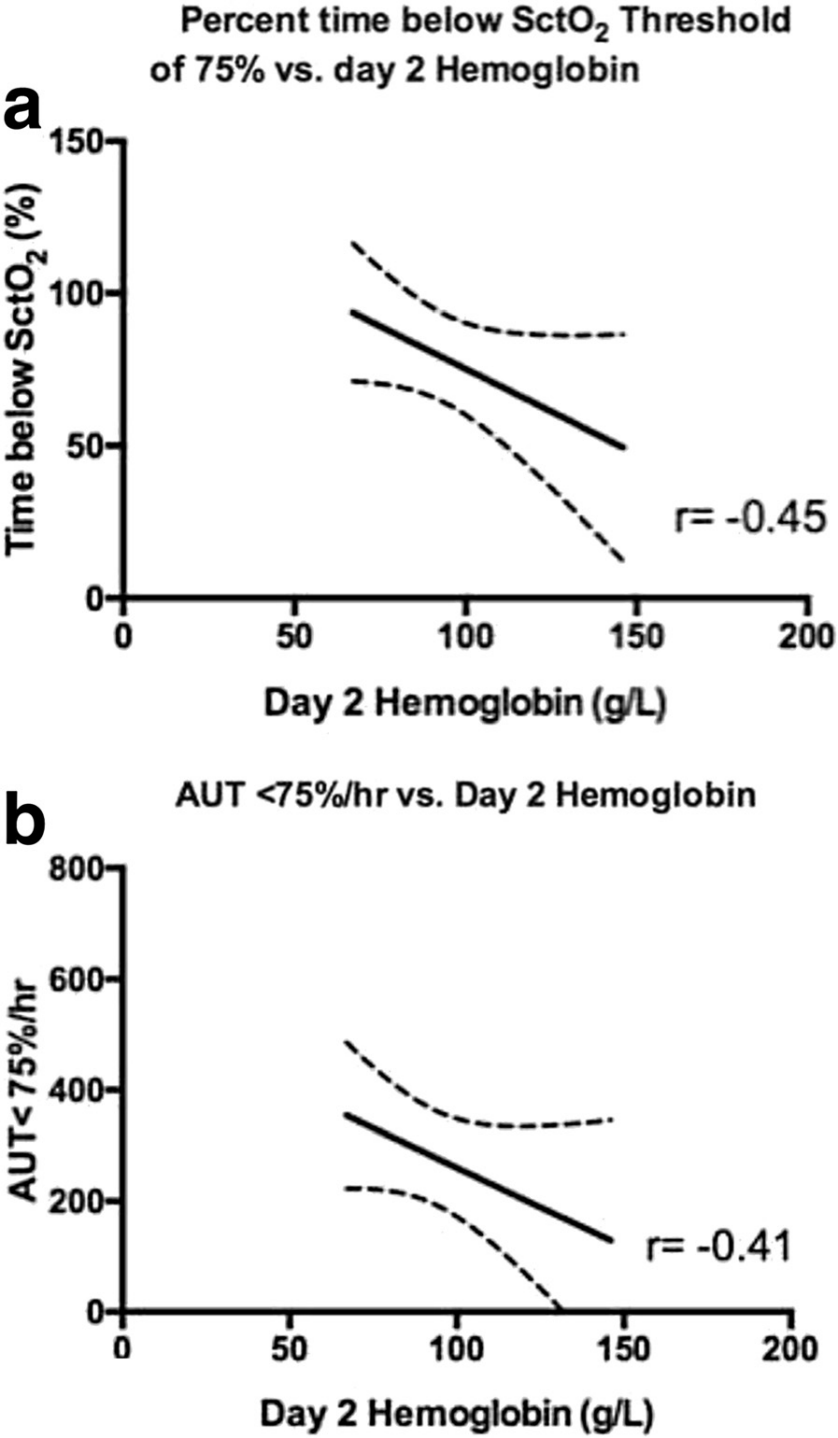
Correlation between **a** percent time below and **b** AUT below a SctO_2_ of 75 % and post intensive care unit admission day 2 hemoglobin. Decreases in hemoglobin concentration were correlated with both the duration (Pearsons *r* = −0.46) and magnitude (Pearsons *r* = −0.41) of cerebral desaturations

With respect to the variables that may affect cerebral blood flow (and therefore potentially cerebral saturation), there was no correlation between S_ct_O_2_ and peripheral oxygen saturation, norepinephrine dose, mean arterial pressure (MAP), or fraction of inspired oxygen (F_I_O_2_) (Fig. 2a–c). However, there was a correlation between day 2 hemoglobin concentrations and both the percentage time below an S_ct_O_2_ (Pearsons *r* = −0.46, Fig. 3a) and SctO_2_ AUT < 75 % (Pearsons *r* = −0.55, Fig. 3b), with lower hemoglobin values being associated with more significant cerebral desaturations.

As a comparison group, we also looked the incidence and magnitude of decreases of cerebral saturation in 30 patients undergoing high-risk non-cardiac surgical procedures, a group known to be at high risk for decreases in SctO_2_. The surgical patients were significantly older (71 ± 6 vs. 57.0 ± 3.6 years, *p* < 0.001) and were coming for a variety of high-risk procedures (see Table 2).

Nine of the surgical patients (30 %) experienced a decrease in their SctO_2_ to below 65 %. Patients in the septic shock group were significantly more likely to experience a cerebral desaturation than those in the surgical group (12/15 vs. 9/30; relative risk 2.7 [95 % CI 1.5–4.9], *p* < 0.01; Table 4). The patients with septic shock also spent more time below an SctO_2_ threshold of 65 % when compared with the patients undergoing high-risk surgery (2.2 % [0.9–10.9] vs. 0.0 % [0.0–8.8], *p* = 0.03, septic vs. surgical patients, respectively, see Table 4). The septic patients also had a greater magnitude of cerebral desaturation (based on AUT calculation) when compared with the surgical patients (3.1 [0.3–14.5] vs. 0.0 [0.0–6.9] % min^−1^ hr^−1^; *p* < 0.05, septic vs. surgical, respectively, see Table 4).

**Table 4:** Comparison of cerebral saturation between septic and surgical patients

Parameter	Septic	Surgical	*p* value
Number of patients with desaturations	12/15	9/30	< 0.01
Percent time below SctO_2_ threshold	2.2 [0.9–10.9]	0 [0.0–8.8]	0.03
AUT/h	3.1 [0.3–14.5]	0.0 [0.0–6.9]	0.04

Septic patients had decreases in their SctO_2_ more frequently and of a greater magnitude than surgical patients. Data are presented as median [interquartile range]. The number of patients with desaturations was compared using Fisher’s exact test, while percent time and AUT/Hr differences were compared with Mann-Whitney *U* test

*Percent time* percentage of time spent below an SctO_2_ of 65 %

*AUT* area under threshold (% min^−1^ hr^−1^)

## Discussion

Our study did not demonstrate any relationship between decreases in SctO_2_ and the incidence of delirium in patients admitted to the ICU with septic shock. We chose the S_ct_O_2_ threshold of 65 % as constituting a significant cerebral desaturation, based on work by other authors [10, 22–24]. We also did not demonstrate any relationship between decreases in SctO_2_ and the development of an AKI. We did however discover that patients who died from their septic shock had significantly larger decreases in their SctO_2_ compared with those who survived.

The lack of an association between decreases in SctO_2_ and delirium could potentially be due to several factors. First, the etiology of delirium is multifactorial, and exact causes of it are uncertain. Second, most of our patients received benzodiazepines, a class of drugs that has been associated with postoperative delirium [25]. Finally, the cerebral oximeter we used only measures SctO_2_ from the frontal lobes and would not detect desaturations that take place in deeper cortical structures. Thus, we might be missing changes in SctO_2_ that occur in deeper parts of the brain that could predispose patients to delirium.

In our study, patients who died from septic shock had larger decreases in their SctO_2_ when compared with those patients who survived. Due to the small number of patients studied, it is possible that this is a spurious finding. There is biological plausibility, however, for decreases in SctO_2_ being linked to death.

Animal studies have demonstrated that as global oxygen delivery is reduced, blood flow and therefore oxygen delivery to the brain is preserved [15]. This may explain the association between decreases in SctO_2_ and non-neurologic organ injury. The brain can be considered the organ of highest priority when it comes to tissue hypoperfusion during shock states. When oxygen delivery to the brain is decreased below a critical value, cerebral desaturations occur. In the context of septic shock, cerebral desaturation likely indicates that blood flow to the other vital organs has been severely compromised. Our work suggests that non-survivors of septic shock exhibit decreased SctO_2_ as a reflection of more severe global perfusion defects compared to survivors. Our study is obviously too small to confirm this, but is hypothesis generating.

When compared with a group of high-risk patients undergoing major non-cardiac surgery at our institution, patients with septic shock had a higher incidence and greater magnitude of cerebral desaturations. We chose high-risk surgical patients as a comparator group as it is known that these patients have high rates of cerebral desaturation and are at high risk for complications. The incidence of cerebral desaturations in our surgical patients is consistent with previous studies that showed a cerebral desaturation incidence of 20–30 % in patients undergoing elective non-cardiac surgery [10, 12, 17, 22, 23]. Our findings suggest that cerebral desaturations are more common and more severe in septic shock patients.

Other interesting findings from our study are the lack of relationship between SctO_2_ and peripheral oxygen saturation, F_I_O_2_, MAP, or vasopressor dose. With the knowledge that decreases in SctO_2_ are related to the outcome, there have been proposed treatment strategies to increase SctO_2_ in the hopes of improving the outcome [26]. These strategies include increasing MAP, increasing arterial carbon dioxide tension (to increase cerebral blood flow), increasing F_I_O_2_, and increasing hemoglobin concentration with red blood cell transfusions. Our work suggests that, in septic patients, these variables (with the exception of hemoglobin concentration) do not correlate with cerebral saturation.

Other perioperative studies have pointed to anemia as a potential cause of adverse neurological outcomes [27]. However, the literature regarding the beneficial effects of blood transfusion in critical care is controversial, with the most recent study suggesting no benefit of higher hemoglobin levels in septic patients [28–30].

Limitations to our study include the small number of patients studied and the varying etiologies of sepsis and the different baseline co-morbidities. A much larger study will have to be undertaken to determine if there is truly no relationship between cerebral desaturations and delirium.

Finally, the issue of extracranial contamination might have affected our results [31]. Briefly, extracranial contamination occurs when desaturated blood below the skin is measured by the device and interpreted as being intracranial in nature. With the aggressive fluid resuscitation that our patients were receiving, it is possible that the edema that results from fluid administration might have resulted in spuriously lower levels of measured SctO_2_.

## Conclusions

Our study suggests that decreases in SctO_2_ occur frequently in patients with septic shock. These decreases are of a larger magnitude than in a group of patients undergoing high-risk non-cardiac surgery. Decreases in SctO_2_ are not related to ICU delirium, but may be related to the risk of death in the ICU. Finally, there appears to be no relationship between the incidence or magnitude of decreases in SctO_2_ and physiological parameters (such as F_I_O_2_, MAP, or vasopressor dose). However, decreases in hemoglobin concentration appear to correlate with changes in SctO_2_.

## Ethics approval

This report describes human research. IRB contact information: Bannatyne Research Ethics Board, P126 Pathology Building, 770 Bannatyne Avenue, University of Manitoba, Winnipeg, MB R3E 0W3. Phone: 204 789-3255. Fax: 204 789-3414. This study was conducted with written informed consent from the study subjects. The study was registered prior to patient enrollment as B2011:138. Registry URL: ClinicalTrials.gov Identifier: NCT1836302. Registered on April 16, 2013.

## Abbreviations

AKI, acute kidney injury; APACHE II, Acute Physiology and Chronic Health Evaluation II; AUT, area under threshold; CAM-ICU, Confusion Assessment Method for the Intensive Care Unit; F_I_O_2_, fraction of inspired oxygen; MAP, mean arterial pressure; SctO_2_, brain tissue oxygen saturation; SOFA, Sequential organ failure assessment
